# Nanoencapsulation of B-toxin from herbal extracts: Targeting HTLV-1 protease and ATLL

**DOI:** 10.22038/ijbms.2025.83900.18154

**Published:** 2025

**Authors:** Arezoo Baghban, Mohammad Momen Heravi, Seyed Abdolrahim Rezaee, Mohsen Tafaghodi, Mohammadreza Bozorgmehr

**Affiliations:** 1 Department of Chemistry, Mashhad Branch, Islamic Azad University, Mashhad, Iran; 2 Immunology Research Center, Inflammation and Inflammatory Diseases Division, Faculty of Medicine, Mashhad University of Medical Sciences, Mashhad, Iran; 3 Nanotechnology Research Center, School of Pharmacy, Mashhad University of Medical Sciences, Mashhad, Iran

**Keywords:** Cancer, Human T-cell leukemia - virus type 1 (HTLV-1), Nanoparticles, Paclitaxel, Poly lactic-co-glycolic acid - (PLGA), Retrovirus

## Abstract

**Objective(s):**

Toxin B and isotoxin B (TB, isoTB) are major constituents of the *Taxus baccata *tree. This study investigates the inhibitory effect of TB and isoTB on adult T-cell leukemia/lymphoma (ATLL), particularly on human T-lymphotropic virus type 1 protease (HTLV-1 PR). HTLV-1 protease (HTLV-1 PR) is an aspartic acid protease and a promising therapeutic target for human immunodeficiency virus (HIV) PR inhibitors.

**Materials and Methods:**

The anticancer properties of *T. baccata* plant components encapsulated in PLGA nanoparticles (NPs/ PLGA/TB) were evaluated by *in vitro *assays using different cell lines. Cancerous cell lines, including HTLV-1-infected-MT2, were treated with varying concentrations of TB and alcoholic extract, and a combined peptide was designed and expressed using recombined NPs/PLGA/TB in a human Fc gamma1 (HTLV-1 PR: hFc gamma1) against HTLV-1.

**Results:**

Our results show that the viability of cancer cells after NPs/ PLGA/TB treatment significantly decreased in a time- and dose-dependent manner using the MTT assay. The inhibitory effect of NPs/ PLGA/TB on the HTLV-1-infected-MT2 cell line, in the absence of recombinant peptide, was (38.98 ± 0.23) and in the presence was (16.18 ± 2.03) in 72 hr (*P*<0.001). This indicates a double inhibitory effect in the presence of the peptide. The enzymatic effect of HTLV-1-protease on its fluorochrome substrate in the presence of TB and isoTB showed nearly complete enzyme inhibition.

**Conclusion:**

These findings present a promising avenue for introducing therapeutic agents with anticancer properties to treat progressive cancers, such as viral ATLL, and inducing effective antiviral responses.

## Introduction

Plants contain a wide variety of phytochemicals that are beneficial to human health. Phytochemical compounds have been shown to have many biological activities, including anticancer, anti-inflammatory, anti-oxidant, and antimicrobial effects (1). *Taxus baccata* contains numerous toxic compounds, including nitriles (cyanogenic glycoside esters) (2), ephedrine ,and its essential oil Toxin B and isotoxin B (TB, isoTB) are the major constituents of *Taxus baccata* tree (3).

HTLV-1 is an RNA virus of the genus Delta-retrovirus, family *Retroviridae*. Historically, this virus is known as the first human oncovirus. HTLV-I causes two life-threatening diseases, including HTLV-1-associated myelopathy/spastic paraparesis (HAM/TSP) as a neurodegenerative disease and adult T-lymphoma/leukemia (ATLL) (4). HTLV-1 protease (PR)cleaves the viral gag/pro-pol polyproteins during maturation and has essential roles in viral replication. In another member of retroviruses, HIV-1, anti-PR is one of the main antiretroviral agents in AIDS treatment. Like HIV-1 PR, HTLV-1 PR is an aspartyl proteinase homodimer (5). Like HIV-1 PR, HTLV-1 PR is an aspartyl proteinase homodimer. HTLV-1 PR shares 28% sequence identity with HIV-1 PR, the substrate binding region, but HIV anti-protease cannot inhibit HTLV-1 protease, so they have different substrate specificities (6). Therefore, for the control of HTLV-1 infection, anti-PR could presumably have such an impact (7, 8).

Taxanes are a group of toxic compounds in the diterpene family. They were first extracted from the European yew (*Taxus baccata*). The antineoplastic drug paclitaxel is commonly extracted from the European yew. It is prescribed under the brand name Taxol for the treatment of many types of cancer. Furthermore, it is possible to enhance the effect of therapeutic agents by precise delivery to the tissue of interest. The biodegradable polymeric nanoparticles, such as PLGA and some single particles, can be used for this purpose and are highly preferred over other carriers. The antineoplastic drug paclitaxel is commonly extracted from the European yew. It is prescribed under the brand name Taxol for treating many cancers (9-11).

In addition, they are not mutagenic and should not cause secondary malignancies or accelerate the progression of benign malignancies. 

Cancer, which is characterized by abnormal growth and uncontrolled proliferation of cells, is one of the leading causes of human death worldwide each year. Although it has become much easier to treat, it remains one of the most serious diseases threatening human health. Certain types of cancer remain challenging to treat because it is difficult to target and deliver drugs to specific cancer cell types. Surface proteins expressed on the surface of malignant cells are increasingly defining human cancers. Selective targeting of cells using these surface proteins is inherently different from surgery, radiation, and chemotherapy and is often considered a new modality for cancer therapy (12).

However, to reach intracellular targets and avoid diffusion to unwanted tissues, these therapies require specific cell targeting mechanisms to deliver the drug cargo across the cell membrane efficiently. Conventional drug delivery systems have limited ability to cross cell membrane barriers, requiring high doses with side effects and safety concerns. In the case of microbial-associated cancers, such as ATLL induced by HTLV-1, targeting HTLV-1 can not only eliminate the viral infection. However, it can also be a therapeutic agent for viral-associated diseases such as ATLL and HAM/TSP (13).

To date, no significant inhibitor of HTLV-1 has been developed. In line with recent research on HIV-1 PR inhibition, new anti-protease agents are needed. We decided to investigate the importance of HTLV-1 PR using TB, isoTB, a potent and selective inhibitor of HTLV-1 infected cancer cells (MT2 cell line), to modify the protease binding site of substrate-ligand interactions of HTLV-1 PR. Three cancer cell lines will also be studied to compare these compounds’ anticancer and antiviral activities. These cell lines include MCF-7 (mammary adenocarcinoma), A549 (human alveolar basal epithelial cell adenocarcinoma), and HT29 (human colon cancer cell line). The toxicity and fluorescence studies of TB, isoTB on cancer cell lines, and HTLV-1 protease enzyme activity were investigated. After confirming the inhibitory effect of TB, isoTB on PR, they were prepared using a delivery system, the technology of plant synthesis (PLGA), using paclitaxel PLGA nanoparticles as a positive control.

## Materials and Methods

### Standards and chemicals

Poly-vinyl alcohol (PVA, MW 146,000–186,000), poly (l-lactide) (PLA, MW 75,000), poly (d,l-lactide-co-glycolide) (PLGA 50:50, MW 75,000 and 5000 as well as PLGA 75:25, MW 75,000 and 5000),  D-ɑ-tocopheryl polyethylene glycol succinate (vitamin E TPGS). The drug that was chosen as the reference standard is the anticancer drug PACLITAXEL PARENTERAL INJECTION 6 mg/1ml by AqVida GmbH (Kaiser-Wilhelm-Str. 98-20355 Hamburg, Germany). Human cancer cell line MCF-7 A549, HT29, MT2 was bought from the Pasteur Institute. HPLC grade acetonitrile and water were obtained from VWR Chemicals (Darmstadt, Germany), and dichloromethane was obtained from Merck (Steinheim, Germany). Ammonium format was obtained from Sigma-Aldrich (Steinheim), and formic acid was obtained from VWR Chemicals (Darmstadt, Germany). Ammonium buffer (pH 9) consists of ammonium hydroxide and ammonium chloride.

### Nanoparticle preparation

Nanoparticle production techniques affect the properties and morphology of synthetic nanoparticles, such as porosity, particle size, stability, shape, and size distribution(14). The most common method in fabricating a PLGA-based nano-sized drug delivery system was utilized in this study, and some modifications were made. Briefly, PLGA (100 ± 5 mg) was poured into a 13 mm × 100 mm test tube, and then dichloromethane (DCM) or ethyl acetate (EtAc) (1.0 ml) was added using a glass serological pipette. The mixture is left overnight. Vitamin E-TPGS solution (0.3% w/v, 45.0 ml) was poured into a beaker and stirred at 360 rpm using a magnetic stir bar. Vitamin E-TPGS solution (2.0 ml) was added to the PLGA mixture. The mixture was vortexed for 20 sec and sonicated for 10 min using an ultrasonic probe (700 W at 40% amplitude) in an ice water bath in triplicate. During the time between ultrasonication, the resulting emulsion was cooled in an ice-water bath. The Vitamin E-TPGS solution (0.3% w/v, 1-2 ml) was added to the emulsion before the nanoparticles hardened under stirring for four hours. Deionized water (15.0 ml) was added to the pellet of nanoparticles and placed in an ultrasonic bath to resuspend the nanoparticles (15).

 The hardened nanoparticles were then loaded into Oak Ridge centrifuge tubes (30 ml nominal volume) and balanced to within 0.1 g. The nanoparticles were centrifuged in a fixed-angle rotor for ten minutes at 14,000 rpm. The supernatant was discarded, and then 15 ml of diH2O was added. A water bath sonicator and vortex were used to resuspend the nanoparticles thoroughly. The washed contents suspended in 30 ml diH_2_O and 4-5 ml of the liquid suspension transferred to new tubes for lyophilization. Finally the lyophilized tubes was stored at -80 °C (16, 17).

### Gas chromatography-mass spectrometry (GC-MS) analysis

**Figure 1:**
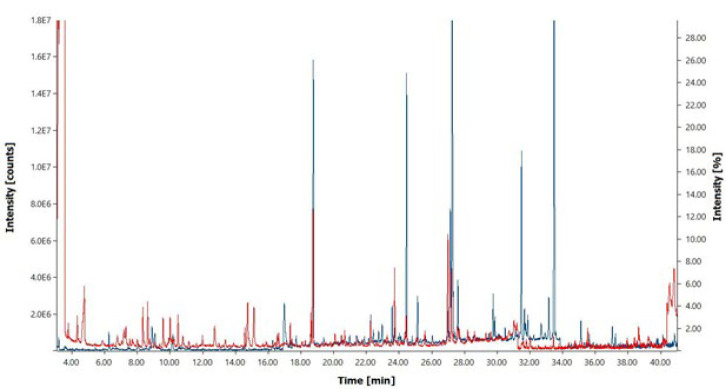
Diagram of GC-MS chromatography, obtained from the alkaloid extract of the* Taxus baccata* tree was obtained with the wiley7n.l mass spectral library, for each compound identified, the retention time (R.T.), Peak Area %. comparing the spectrum of alkaloid extract in a pure state with blue and the state locked in polymer with red color.

The alkaloid compounds had 59 peaks in the GC-MS chromatogram. In this study, we used GC-MS as an innovative method to demonstrate the entrapment of extract particles in PLGA polymer. In peaks 48 and 51, PLGA polymer fragments appear. To verify the polymerization, we compared the GC-MS chromatogram of the pure and polymerized extract (18). This is an innovative method for such analyses. Briefly, samples were analyzed on a gas chromatograph (Varian CP-3800 series) coupled to a mass spectrometer (Varian 1200 L triple-quadrupole) as a continuation of an autosampler (CTC COMBI PAL, Varian Inc., Palo Alto, CA, USA). Sample chromatography was performed using a guard column (5 m, 0.25 mm, 0.25 μm), Restek Corp. Helium was selected as the carrier gas with a flow rate of 1.2 ml/min and a column pressure of 13.2 psi. The total run time was 40 min. The injector temperature and injection volume were 280 °C and 1.0 μl in splitless mode. Samples were analyzed using an ion source temperature of 240 °C and a transfer line temperature of 300 °C. The electron multiplier voltage was set to 1400 V by automatic tuning. Argon as a collision gas and a collision cell of 1.8 m Torr were selected for the MS experiments, and the main procedures were explained (19). Table 1 summarizes the precursor ions determined and their collision energies. Dichloromethane (1.0 ml) was added to the obtained polymer extract, followed by vortexing for two minutes and centrifugation at 14,000 rpm for 5 min after 1.0 μl of the organic phase was separated and injected. Mass Spectral Library Ver. 3.4. (Figure 1) (20, 21).

### HPLC chromatograph

**Figure 2:**
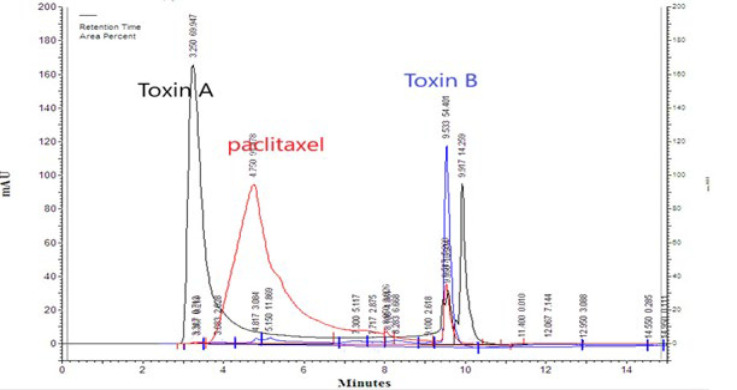
Diagram of HPLC chromatography purification of alkaloid extract (method 1,2) compared with paclitaxel (control) comes out from the *Taxus baccata *tree. The y-axis of the chromatogram is a measure of the intensity of absorbance (in units of mAU or milli-absorbance Units). The x-axis is in units of time (typically minutes) and is used to determine the retention time (tR) for each peak.

The HPLC system (Agilent 1100 series, USA) with a diode array UV detector was chosen to determine and compare Taxol contained in the standard solution (taxol 6 mg/ml - paclitaxel Aq Vida) and extracts of *T. baccata*. L tree bark, stems, and leaves. All analyses of the samples were carried out on an Agilent Eclipse XDB-C18 column (150 × 4.6 mm, 3.5 μm). The mobile phase contains water and acetonitrile in a ratio of 50:50% v/v and a flow rate of 1.2 ml min-1. The column temperature and detector were maintained at 40 °C and 227 nm (22). The injection volume was 20 μl, and Figure 2 shows the separation conditions of the extract and a typical chromatograph (23).

### PLGA NPs characterization

 A dynamic light scattering technique was applied to study the hydrodynamic diameter of PLGA nanoparticles. Each nanoparticle (1 mg) was poured into an Eppendorf tube with a cap before adding chloroform (1 µl) to investigate the extract entrapment. The NPs/ PLGA/TB, NPs/ PLGA alcoholic *T. baccata* extracts and NPs/ PLGA/paclitaxel were wholly dissolved in chloroform, followed by evaporating chloroform under N2 gas. Methanol (70% v/v, 200 µl) was added to the residue to dissolve the PLGA extract. The supernatant was finally analyzed using an LC-MS (Waters Micro mass ZQ400) based on a previously published article (24). Drug loading (DL) and encapsulation efficiency (EE) were determined using the following equations, and the results are presented in Table 2.

$\mathrm{Eq.}\left(\mathrm{A.1}\right):\mathrm{EE\%}=\frac{\mathrm{Amout of extrat I loaded in}(\mathrm{\mu g})}{\mathrm{Total amount of extract I used}(\mathrm{\mu g})\times 100}$

$\mathrm{Eq.}\left(\mathrm{A.2}\right):\mathrm{DL}(\frac{W}{W})=\frac{\mathrm{Amout of extrat I loaded in}(\mathrm{\mu g})}{\mathrm{Total amount of PLGA in}(\mathrm{\mu g})}$

To mitigate the effects of drug toxicities, complex nanoparticles of PLGA have been prepared that have the advantage of reduced body toxicity and targeted delivery (25, 26). PLGA is ideal for drug delivery applications because its unique polymer structure confers several desirable properties. PLGA is also a suitable coating material. It is often used to increase the solubility and stability of hydrophobic materials. 

### Sizing and surface morphology

The nanoparticles were poured into a centrifuge tube and frozen at -80 °C for 40 min. The valve of the tube was covered with laboratory tissue before the contents of the tube were thawed. The lyophilized particles were frozen in a tube wrapped with parafilm at -80 °C. The polymer may be added at this stage as a cryoprotectant (freeze a small number of lactose-free nanoparticles for SEM imaging) (27). Zeta potential is an essential and easy-to-measure index for the stability of colloidal dispersions. The magnitude of zeta potential indicates the degree of electrostatic repulsion between adjacent and similarly charged particles in a dispersion. Therefore, colloids with a high zeta potential (negative or positive) are electrically stabilized. In contrast, colloids with a low zeta potential tend to fall off or have high fallout, as shown in Table 2 (28). The Polydispersity Index (PdI) approximates average particle solution uniformity, and larger PdI values ​​correspond to a more significant size distribution in the particle sample. PdI can also indicate the aggregation of nanoparticles along with the stability and efficiency of particle surface circulation throughout the particle sample (29).

### MTT assessment

The study assessed the cytotoxic impact of the NPs/ PLGA/TB, NPs /PLGA / alcoholic Taxus baccata extracts and PLGA/paclitaxel nanocomplex (NPs/ PLGA/paclitaxel) on (MCF-7, A549, HT29, and MT2) through an MTT assay, following a previously described protocol. Cells from all four lines in the logarithmic growth phase were seeded into a 96-well culture plate and incubated for 24, 48, and 72 hr. Treatment concentrations of the study components ranged from 0 to 300 ng μl-1. A flow cytometer from BD Biosciences (BD Biosciences, San Jose, CA, USA) was utilized to analyze and quantify cell numbers (30). The NPs/ PLGA/paclitaxel was tested at concentrations of 0 (control negative group), 120, 180, 240, and 300 ng μl-1 over 24-, 48-, and 72-hr intervals on the A549, MT2, HT29, and MCF7 cells to determine cell survival rates. Absorbance values were normalized by dividing the mean absorbance of treated wells by that of control wells containing only cells and culture medium (negative control). Paclitaxel was used as a positive control, with the anticancer drug paclitaxel from AqVida GmbH (Germany) serving as the reference standard. All experiments were conducted in triplicate. All treatments were adjusted and evaluated simultaneously to eliminate the effect of variation.

### Statistical analysis

It is important to note that all experimental protocols described in this study were carefully replicated at least three times. Analyses were performed in triplicate. Statistical analyses were performed using GraphPad Prism (version 9.5.1, GraphPad, USA) and SPSS version 11.5 (SPSS, Chicago, IL, USA). Collected data were presented as mean ± standard deviation (SD). Pearson’s correlation coefficient was used for correlational analyses, and one-way ANOVA with Tukey’s post-test was used for statistical analyses between groups, with significance set at *P*<0.05 (24, 27-29).

## Results

### Toxicity analysis

**Figure 3:**
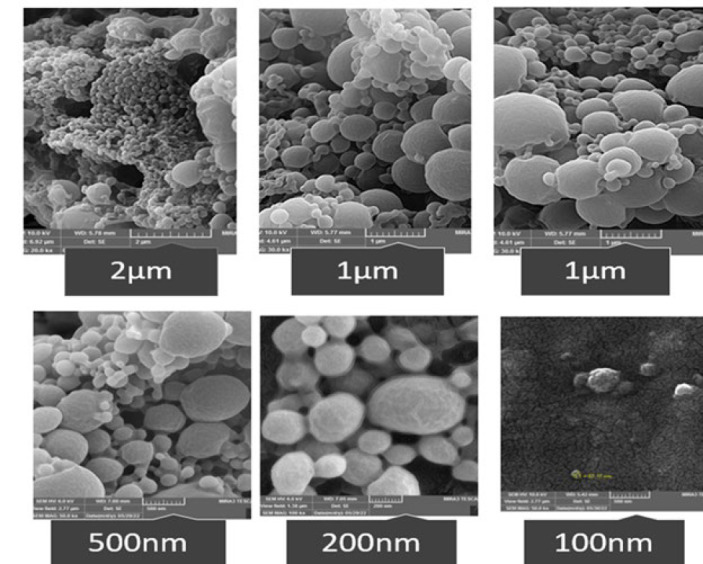
Examining the size of The NPs/ PLGA/TB, NPs/ PLGA alcoholic *Taxus baccata *extracts, and NPs/ PLGA//paclitaxel in different scales with a scanning electron microscope (SEM).

**Figure 4:**
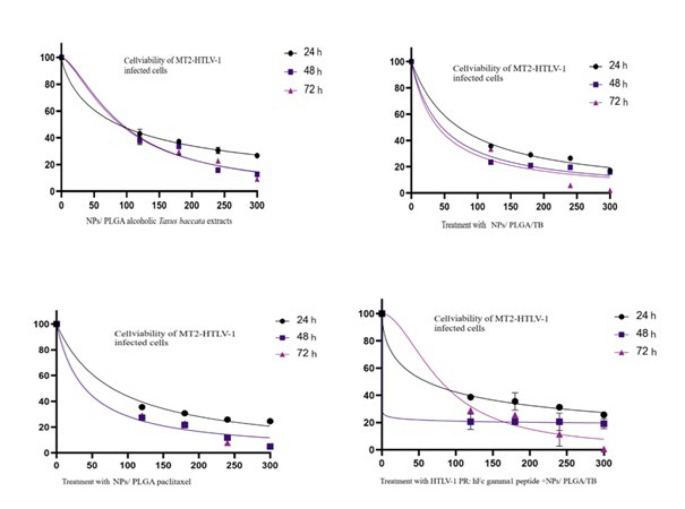
Diagram of survival fraction MT2 (MT2-HTLV-1 infected cells) in different concentrations with treatment by The NPs/ PLGA/TB, NPs/ PLGA alcoholic *Taxus baccata *extracts, and NPs/ PLGA//paclitaxel in 3 consecutive days. *P*<0.001 compared with control (one-way ANOVA, followed by Tukey's test). Mean normalized splicing efficiency is displayed as bars, with experimental replicate values represented by different shapes and linked to illustrate the difference in cell rescue.

The toxicity assessment of The NPs/ PLGA/TB, NPs/ PLGA alcoholic *Taxus baccata* extracts and NPs/ PLGA/paclitaxel were conducted for all cancerous cell lines by examining the percentage of viable cells after 24, 48, and 72 hr as recommended (31). Although, in 24, 48 hr significant effects were observed, the maximum impact was in 72-hr incubation. The survival rates of cells exposed to varying concentrations of the NPs/ PLGA//paclitaxel (ranging from 0 to 300 ng per microliter) across multiple cell lines were presented in Table 4 for different treatment times and in Figure 3 and 4 only for HTLV-1-MT2 cell line. It could be noted that the toxicity levels of all components were previously assessed (data not shown). TB’s most pronounced inhibitory effect was observed on the HTLV-1-MT2 cell line, with a survival rate of up to 15.92±0.21. The A549 cell line exhibited a survival rate of 39.74±1.06, the HT29 cell line 26.45±2.44, and the MCF7 cell line 35.9±1.07 after 72 hr. 

The survival rates of cells exposed to varying concentrations of the NPs/ PLGA alcoholic *T. baccata* extracts (ranging from 0 to 300 ng per microliter) across multiple cell lines were monitored and analyzed over three days, the most pronounced inhibitory effect was observed in the HTLV-1-MT2 cell line, with a survival rate of up to 19.68±1.06, The A549 cell line exhibited a survival rate of 86.76±0.95, the HT29 cell line recorded 86.33±1.75, and the MCF7 cell line displayed 74.38±0.43 survival after 72 hr. The survival rates of cells exposed to varying concentrations of NPs/ PLGA/TB (ranging from 0 to 300 ng per microliter) across multiple cell lines were monitored and analyzed over three days; the most pronounced inhibitory effect was observed in the HTLV-1-MT2 cell line, with a survival rate of up to 38.98±0.23. The A549 cell line exhibited a survival rate of 42.99, the HT29 cell line 40.09±2.01, and the MCF7 cell line 40.8±0.10 after 72 hr. The survival rates of cells exposed to different concentrations of PLGA/ HTLV-1 PR: hFc gamma1 peptide + NPs/ PLGA/TB (ranging from 0 to 300 ng per microlitre) in several cell lines were monitored and analyzed over 3 days; the most pronounced inhibitory effect was observed in the HTLV-1-MT2 cell line, with a survival rate of up to 16.18±2.03 after 72 hr (*P*<0.001). The comparative results of the investigated treatments are shown in Table 4.

### Sizing and surface morphology

The morphology of the dried complex of The NPs/ PLGA/TB, NPs/ PLGA alcoholic *T. baccata *extracts and NPs/ PLGA//paclitaxel were examined by SEM. Similarly, PLGA particles have non-melting spheres of different sizes and smooth surfaces (32). The average particle size in PLGA NPs/ alcoholic samples was 178.9 nm, NPs/ PLGA/TB 151.9 nm, and NPs/ PLGA//paclitaxel 146 nm, and for Lactose monohydrate, it was 145.5 nm. A particle size between 100 and 300 nm is suitable for a drug delivery system (Figure 3). The encapsulation efficiency (EE) (Eq. (A.1) was 85.8, and the drug loading (DL) (Eq. (A.2) was 12.5 (Table 3). The zeta potential nanoparticles of lactose monohydrate alone and NPs/ PLGA/TB, NPs/ PLGA alcoholic *T. baccata *extracts and NPs/ PLGA//paclitaxel were assessed for 30 min at pH 7 (Table 3) (33). The zeta potential of lactose monohydrate nanoparticles was -4.71 ± 3.64 mV, indicating weak dispersion stability. The zeta potential of NPs/ PLGA / alcoholic (-3.68±4.53 mV), NPs/ PLGA/TB (-6.67±7.95 mV), and NPs/paclitaxel were (-11.3 ± 3.54) mV, indicating that it has average dispersion stability (Table 2). Nanoparticle analysis results confirm that PLGA nanoparticles have the appropriate size and excellent ability to selectively deliver drugs to the desired site in the human body. The SEM images showed a particle size of 85.8 nm, consistent with the necessary (80 nm-146 nm) size. The polydispersity index (PDI) is considered as a parameter to realize the particle size distribution of nanoparticulate (11.3 ± 3.54), which indicates the particle size distribution; the stability of nanoparticulate is low as suggested (34). 

### Protein structure quality determination

**Figure 5:**
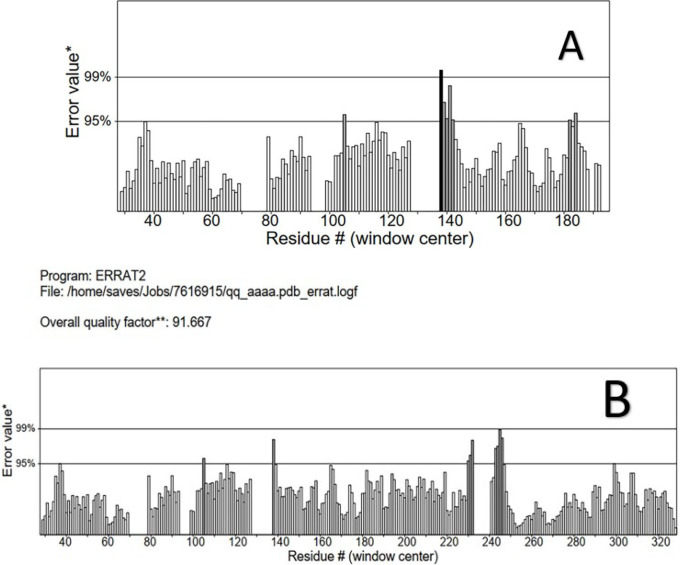
ERRAT plots for (A) HTLV-1 PR; (B) HTLV-1 PR with hFc gamma1 peptide mutants

The protein structures were checked for quality using other structure verification tools like ERRAT. I (35). The ERRAT score for HTLV-1 PR (A) was the highest (94.161%) and very close for the HTLV-1 PR with hFc gamma1 peptide (91.667%) (B in Figure 5) (36). The results showed that the backbone structure, the nonbonded interactions, and the mutants were well within the range of good models. 

### Fluorescent emission assay

**Figure 6:**
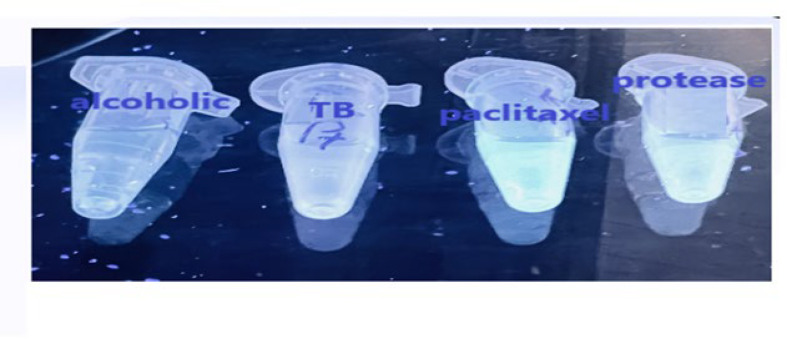
λem and λex of HTLV-1 -PR and substrate were recorded

**Figure 7:**
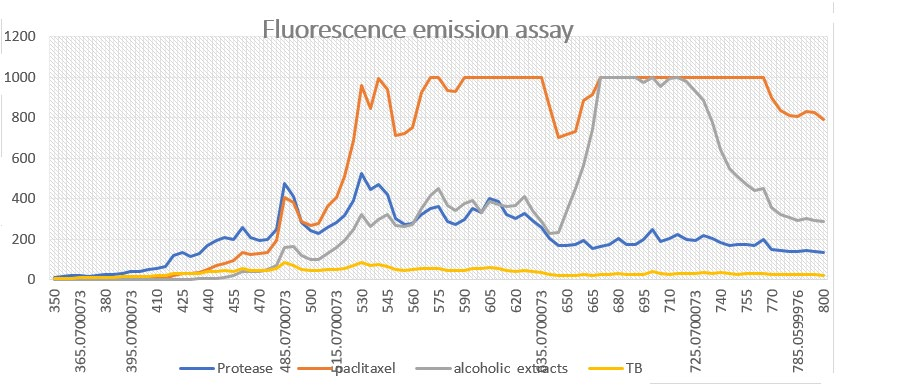
For the emission scans, the excitation wavelength was set at 400 nm, the emission wavelengths were scanned from 400 to 800 nm, and the maximum λem was 490 nm

When cleaved, the selective substrate for HTLV-1 PR, synthesized with the green fluorochrome, emits a wavelength of 490-520 nm in the green region under UV wavelength. The results of the present study showed that the fluorescence emission of the substrate cleaved by HTLV-1-PR was much more inhibited by the TB, alcoholic *T. baccata* extracts, and paclitaxel than by the untreated substrate-HTLV-1-PR mixture (Figure 6). Therefore, it seems that TB and alcoholic extracts of *T. baccata* are potent inhibitors of HTLV-1 protease and can be used as a drug in the future (Figures 6 and 7).

## Discussion

Patients with adult T-cell leukemia/lymphoma (ATLL) can present with a wide range of clinical manifestations, from smoldering and chronic to very aggressive forms such as acute leukemia or lymphomatosis (37, 38). The extremely high mortality rate associated with this disease is often due to a combination of many factors, depending on the sites of involvement, including lymph nodes, liver, spleen, and skin lesions. However, an effective drug for ATLL has yet to be introduced. The MT2-HTLV-1 cell line has the characteristic of ATLL cancer cells, on which, in this study, the effects of our extracts were evaluated and also compared with three different cancer cell lines (MCF-7, A549, HT29) (39, 40).

One of this study’s new and innovative aspects is the extraction of The NPs/ PLGA/TB, NPs/ PLGA alcoholic *T. baccata* extracts and NPs/ PLGA/paclitaxel. To further investigate the cytotoxicity and inhibitory effects of The NPs/PLGA/TB, NPs/PLGA alcoholic *T. baccata* extracts and NPs/PLGA/paclitaxel were evaluated as therapeutic approaches in four cell lines more focused on HTLV-1 ATLL. The chemotherapeutic drug paclitaxel was used as a positive control at all stages of the study. In general the results of this study compare with those generally obtained with pure extracts showed that the anti-cancerous and particularly anti-viral increased and at the same level the toxicity of the components was reduced when encapsulated in forms of NPs/PLGA/TB, NPs/PLGA alcoholic *Taxus baccata *extracts and NPs/PLGA/paclitaxel (41). Disadvantages of conventional drug delivery include internal environmental effects on drug absorption, poor patient compliance, high cost, poor drug bioavailability, low mucosal penetration efficiency, and toxic effects on normal tissues. However, micro- and nanotechnology can address most of the above disadvantages of conventional drug delivery. It has significant advantages in achieving targeted drug delivery, sustained release, enhanced solubility, improved pharmacokinetics, prolonged circulation, and reduced toxic side effects. This is a promising sign that the particles complexed with the carrier can stay in the body for longer and release more drug with less damage to healthy cells. Fortunately, we demonstrated the inhibitory effect in all cancer lines with less than 50% survival after 72 hr (*P*<0.001).

The Fc-fused recombinant peptides are easy to purify, have a long half-life, are more stable, and under certain conditions, the Fc tag can act as a targeted delivery vehicle (42, 43). The HTLV-PR used in this study is an efficient Fc-fused recombinant peptide that cleaves a selective substrate. The results of this study showed that the inhibitory effects of the drug preparations, including NPs/PLGA/TB and alcoholic *T. baccata*, on HTLV-PR were very significant. Figure 6 shows that the positive control (Paclitaxel) failed to inhibit the cleavage of the substrate and potentiated the enzymatic activities of HTLV-1-PR. While NPs/PLGA/TB had potent inhibitory effects on HTLV-1 PR, NPs/PLGA/alcoholic *T. baccata* did not. The inhibitory effects could be due to two different mechanisms: binding to the enzyme’s active site or allosteric modification of the enzyme (44). These findings are inconsistent with the results of these components on the MT2-HTLV-1 cell line, a cancer cell line of viral origin and the main target of the present study. They are also the line with which by comparing the inhibitory results in the absence of peptide (38.98±0.23) and in the presence of peptide (HTLV-1 PR: hFc gamma1) (16.18±2.03) treated with PLGA complex during 72 hr. Other therapies, such as neutralizing monoclonal antibodies, inhibitory cytokines, and combined chemotherapies, have been proposed for aggressive types of ATLL, but the survival rate for such subjects was around 7-11 months (38). This result is promising for the production of combined drugs with anticancer properties for the treatment of cancers such as ATLL of viral origin (45). Although the inhibitory effects of Taxus extract have been the subject of many studies, the encapsulated forms have rarely been evaluated. In this study, the inhibitory effect of pure extracts on protease and cancer cell lines was confirmed in the form of nanoparticles encapsulated on the desired cell lines. The results showed that encapsulation reduced the compounds’ toxicity compared to non-capsulated extracts and increased the inhibitory effect on virus and cancer cell lines. Therefore, it may be more effective under *in vivo* conditions, i.e., increasing the duration of drug retention in the body with less toxic effects. 

According to the results in Table 4, the toxicity reported for paclitaxel at various concentrations within 72 hr is higher. However, the concentration of the original drug, paclitaxel, complexed in nanoparticles is three times higher than that of the extracts. Thus, comparing the results and considering the 3:1 ratio, the inhibitory effect of the extracts is greater than that of the drug paclitaxel. 

Furthermore, the NPs/PLGA/TB and NPs/PLGA alcoholic extracts of *T. baccata* could directly inhibit the HTLV-1 protease in the fluorescence emission assay. Therefore, the NPs/PLGA/TB extract can be used as an antiretroviral drug. The strong effects on HTLV-1 PR showed why these compounds were more effective on the HTLV-1 infected MT2 cell line than on other cancer cells. The study has some limitations; finding the effects of these extracts *in vivo* assessments could give it more strength, but the stigma around this virus in this region make this almost impossible.

## Conclusion

Traditional cancer therapies remain essential for immediate tumor control but are often associated with significant side effects. The future of cancer treatment may lie in integrating these approaches to take advantage of their respective strengths. There is no effective treatment for HTLV-1-associated diseases, HAM/TSP and ATLL, or successful development to find a significant inhibitor against this virus. The NPs/PLGA/TB and NPs/PLGA-alcoholic *T. baccata* extracts were key inhibitors of all cancer cell lines tested. Moreover, at 0.03 ng, 100 times less than the effective dose on cell lines, they could inhibit almost 100% of HTLV-1 PR enzymatic activity in the fluorescence assay. It is more likely that the stronger inhibitory effect of these compounds on the HTLV-1-infected MT2 cell line was related to this activity. 

In general, preparing complex nanoparticles on PLGA scaffolds has the advantage of reduced toxicity and the ability to precisely target the desired site, a key molecular target for cancer therapeutics, particularly the HTLV-1-associated malignancy, ATLL. Further studies in animal and clinical models may establish a combination therapy of peptide vaccination and chemotherapeutic agents. 

**Table 1:** Major Phyto-components and their biological activities obtained through the GC/MS Study of the *Taxus baccata *tree. Alkaloid extract have been listed in the Mass Spectral Library Ver. 3.4.5, for each compound identified, the Retention Time (RT), Peak Area %, Mol. wt. Formula, and compound names are listed

PK	Common name	Peak area %	RT/min	Mol. Wt.	Formula
PK 1	2-Furanol	5.39	3.175 5.39	84.07 g/mol	C_4_H_4_O_2_
PK2	1,3-Cyclopentanediol	29.75	3.347	102.13 g/mol	C_5_H_10_O_2_
PK3	N,1-dimethylpiperidin-3-amine	43.76	3.543	128.22 g/mol	C_7_H_16_N_2_
PK6	gamma-Butyrolactone	1.17	4.325	86.09 g/mol	C_4_H_6_O_2_
PK19	Isobutyl butyrate	0.75	14.739	144.211g/mol	C_8_H_16_O_2_
PK20	2-butyloxolane	0.67	15.130	128.21g/mol	C_8_H_16_O
PK31	1-Hexadecanol	0. 73	23.738	242.44 g/mol	C_16_H_34_O
PK34	1-Octadecanol	1.14	26.992	270.49 g/mol	C_18_H_38_O
PK35	Methyl 6-octadecenoate	0.74	27.215	296.49g/mol	C_19_H_36_O_2_
PK54	Glyceryl mono linoleate	0.83	38.644	354.5 g/mol	C_21_H_38_O_4_
PK58	Tri (ethylene glycol) mono ethyl ether	0.88	40.548	178.23g/mol	C_8_H_18_O_4_
PK59	2-(Benzyloxy)benzaldehyde	1.08	40.823	212.24g/mol	C_14_H_12_O_2_

**Table 2:** Shows the experimental results of Zeta potential measurements at pH 7 for The NPs/ PLGA/TB, NPs/ PLGA alcoholic *Taxus baccata *extracts, and NPs/ PLGA//paclitaxel Size distribution of average particle size and dispersion index obtained from polymer particles.

Type of extract	Average particle size (nm)	PdI	Zeta potential (mV)
Lactose monohydrate	145.5 nm	0.203	-4.71±3.64
NPs/ PLGA alcoholic extract	178.9 nm	0.28	-3.68±4.53
NPs/ PLGA/TB	151.9 nm	0.228	-6.67±7.95
NPs/ PLGA/paclitaxel	146 nm	0.228	-11.3±3.54

**Table 3:** Entrapment efficiency and average drug loading for NPs/ PLGA/TB, NPs/ PLGA alcoholic *Taxus baccata* extracts and NPs/ PLGA//paclitaxel

Type of extract	%EE	% Loading
NPs/ PLGA alcoholic extract	26.715	15.11
NPs/ PLGA/TB	70.75	32.04
NPs/ PLGA /paclitaxel	85.8	12.5

**Table 4:** Cell viability of The NPs/ PLGA/TB, NPs/ PLGA alcoholic *Taxus baccata *extracts, and NPs/ PLGA/paclitaxel against HTLV-1-MT2 cells, A549, HT29, MCF7 cancer cell lines in 24 hr, 48 hr and 300 µg/ml of continuous exposure to each extract.

(Cell viability ⁒ of control)		Treatment with extracts of *Taxus baccata *in 24 hr				Controls		
Cell lines		NPs/ PLGA/TB	NPs/ PLGA alcoholic			Positive (NPs/ PLGA//paclitaxel)	Negative (no treatment)	
HTLV-1-MT2		79.21±132	100±1.66			100±2.21	100 ± 2.03	
A549		100±1.33	100±1.75			100±1.00	100 ± 0.00	
HT29		71.85±2.14	92.89±1.05			74.33±1.44	100 ± 1.14	
MCF7		88.99±1.50	100±1.06			99±1.28	100 ± 5.40	
Values are mean ± SD (n = 6) ∗ = *P*<0.05 a: Extract concentration at 10 µg/ml b: Extract concentration at 20 µg/ml Values are mean ± SD (n = 6) ∗ = *P*<0.05 a: Extract concentration at 10 µg/ml b: Extract concentration at 20 µg/ml								
(Cell viability ⁒ of control)		Treatment with extracts of *Taxus baccata *in 48 hr				Controls	
Cell lines		NPs/ PLGA/TB	NPs/ PLGA alcoholic			Positive (NPs/ PLGA//paclitaxel)	Negative (no treatment)
HTLV-1-MT2		59.46±2.00	43.58±2.38			20.33±1.23	100 ± 2.03
A549		76.12±1.03	92.89±0.35			78.47±1.62	100 ± 0.00
HT29		46.19±1.14	90.06±0.75			31.82±1.20	100 ± 1.14
MCF7		79.01±0.20	98.07±1.19			74.35±0.54	100 ± 5.40
(Cell viability ⁒ of control)		Treatment with extracts of *Taxus baccata *in 72 hr				Controls	
Cell lines		NPs/ PLGA/TB	NPs/ PLGA alcoholic			Positive (NPs/ PLGA//paclitaxel)	Negative (No treatment)
HTLV-1-MT2		38.98±0.23	19.68±1.06			15.92±0.21	100 ± 2.03
A549		42.99±1.03	86.76±0.95			39.74±1.06	100 ± 0.00
HT29		40.09±2.01	86.33±1.75			26.45±2.44	100 ± 1.14
MCF7		40.8±0.10	74.38±0.43			35.9±1.07	100 ± 5.40
Values are mean ± SD (n=3) *P*-value<0.001Extract concentration at 300 µg/ml							
(Cell viability ⁒ of control)	Treatment with HTLV-1 PR: hFc gamma1 peptide +NPs/ PLGA/TB						
Cell lines	224 hr			448 hr	772 hr		
HTLV-1-MT2	73.21±132			40.38±2.00	16.18±2.03		
Paclitaxel, as an anticancer drug currently used in chemotherapy, was tested (negative control:0) at 300 concentrations µg/ml intervals of 24, 48, and 72 hr on MT2 cells infected with HTLV-1 MT2 - HT29 - MCF7 - A549 cells to determine the percentage of cell survival. It should be noted that the treatment is dose and time dependent. Average cell viability (50% inhibition concentration) was determined from percent cytotoxicity. Cytotoxicity of the extract was determined by independent samples Tukey test (*P*-value < 0.001). The study showed that alkaloid and alcohol extracts with positive control (paclitaxel) were effective in inhibiting survival *in vitro*. The results showed that the percentage of cells surviving decreased with increasing time and concentration. Values are mean ± standard errors in triplicate. Values are mean ± Standard Error of triplicate determination.							

Black bars indicate the misfolded region far from the active site, gray bars indicate the error region between 95% and 99%, and white bars indicate the region with a lower error rate for protein folding. * Two lines are drawn on the error axis to indicate the confidence with which it is possible to reject regions that exceed this error value. ** Expressed as a percentage of the protein for which the calculated error value falls below the 95% rejection limit. Good high-resolution structures generally yield values of 95% or higher. For lower resolutions (2.5 to 3 A), the average overall quality factor is around 91%.

The emission spectrum of the complex was measured at room temperature to determine the interaction between enzyme and substrate for evaluating the proper enzyme function, diagram of absorption, and fluorescence spectrum of HTLV-1 -PR in DMSO (λexc = 666 nm)

The results show that the fluorescence emission of HTLV1 PR was much more inhibited by the alcoholic extracts of Taxus baccata TB than by the HTLV1 PR complex. In this situation, by comparing emission, enzyme fluorescence reaction shows that the best inhibition is related to tuberculosis, then alcoholic extracts of Taxus baccata, so it is a good inhibitor for HTLV-1 protease. As shown in the graph, the inhibition rate of the control group (paclitaxel) is much lower than that of the extracts and even higher than that of the protease alone, indicating an increase in protease activity.

## Data Availability

All data generated or analyzed during this study are included in this manuscript and are available from the corresponding author upon reasonable request.

## Declaration

To improve the readability of the paper, the authors used deepl in their writing.
